# An ethnobotanical study of medicinal plants in Sheka Zone of Southern Nations Nationalities and Peoples Regional State, Ethiopia

**DOI:** 10.1186/s13002-020-0358-4

**Published:** 2020-02-05

**Authors:** Zewdie Kassa, Zemede Asfaw, Sebsebe Demissew

**Affiliations:** 1 Department of Biology, Mizan-Tepi University, Mizan Tefer, Ethiopia; 2 Department of Plant Biology and Biodiversity Management, Addis Ababa University, Addis Ababa, Ethiopia

**Keywords:** Diversity, Fidelity, Healthcare, Informant consensus, Phytochemical, Vegetation

## Abstract

**Background:**

People’s classification, management, and use of plants represent attempt to attracting people from different academic disciplines. Many countries use traditional medicine for their primary healthcare system. Medicinal plants have been important components of healthcare systems since the time immemorial. The objective of this research was to document and analyze traditional medicinal plants used by the Sheka people and associated ethnobotanical knowledge.

**Methods:**

Data was collected by administering pre-prepared semi-structured interview items to 414 informants. Market surveys, group discussion, and guided field walk were used. Data were analyzed using descriptive and inferential statistics; determination of informant consensus factor, fidelity level, as well as ranking and scoring.

**Results:**

A total of 266 plant species belonging to 192 genera and 74 families were identified. About 204 (77%) of the medicinal plants were used to treat human health problems. Only ten (4%) were used to treat livestock health problems and 52 (19%) of them were used to treat both human and livestock health problems. Croton macrostachyus, Prunus africana, Peperomia retusa, Lobelia giberroa, and Celosia schweinfurthiana were found to be high ranking medicinal plants against gastrointestinal problems based on simple preference ranking.

**Conclusion:**

Very high number of medicinal plant species recorded from the study area indicates that vegetation of Sheka is reservoir of medicinal plants. Hence, the area needs attention for medicinal plant conservation priorities. Plant parts used as medicines also play vital role in the entire medicinal plant life cycle. Therefore, it is useful to consider harvesting impacts. Except well-experienced traditional healers, people of the study area use the medicinal plants haphazardly. There may be high risk of being victims of dosage and improper usage. High ranking medicinal plants are candidates for further phytochemical profiling, drug research, and development.

## Background

According to Martin [1], the study of people’s classification, management, and use of plants or more simply the science of ethnobotany is an endeavor which attracts people from various academic disciplines. Hence, ethnobotany is the study of the interrelationships between people and plants, particularly the way in which plants impact on human culture, and practices and how humans have used and modified plants, and how they represent them in their systems of knowledge. These relationships can be social, economic, symbolic, religious, commercial, and artistic practices [2–5].

### Medicinal plants

According to [6, 7], many countries use traditional medicine for their primary healthcare system; Ethiopia (90%), Benin (80%), India (70%), Rwanda (70%), Tanzania (60%), Uganda (60%), China (40%), and Africa total (80). From historical perspectives, [8] noted that the establishment of traditional medicine in countries like China is based on thousands of years of experiences owing to prescriptions, principles, and reflections on the human-nature relationships. It was underlined that medicinal plants have been cornerstones of healthcare systems since immemorial times probably over 4000 years [9]. However, from the global perspective, there is lack of complete information on traditional herbal medicine that is collected and stored in databases for global use for the establishment and development of research programmers. Osuki [10] summarized that traditional medicine in Africa has remained an enduring future of the family in particular and of the African society in general. Studies on Ethiopian medicinal plants showed that herbal extracts have been attracting scientific interest due to their potential as sources of phytochemicals against pathogenic microorganisms. Moreover, they play important role in meeting the primary healthcare needs of society [11–14]. Hence, well-documented Ethiopian traditional medicinal plant database is important for drug research.

## Methods

**Fig. 1:**
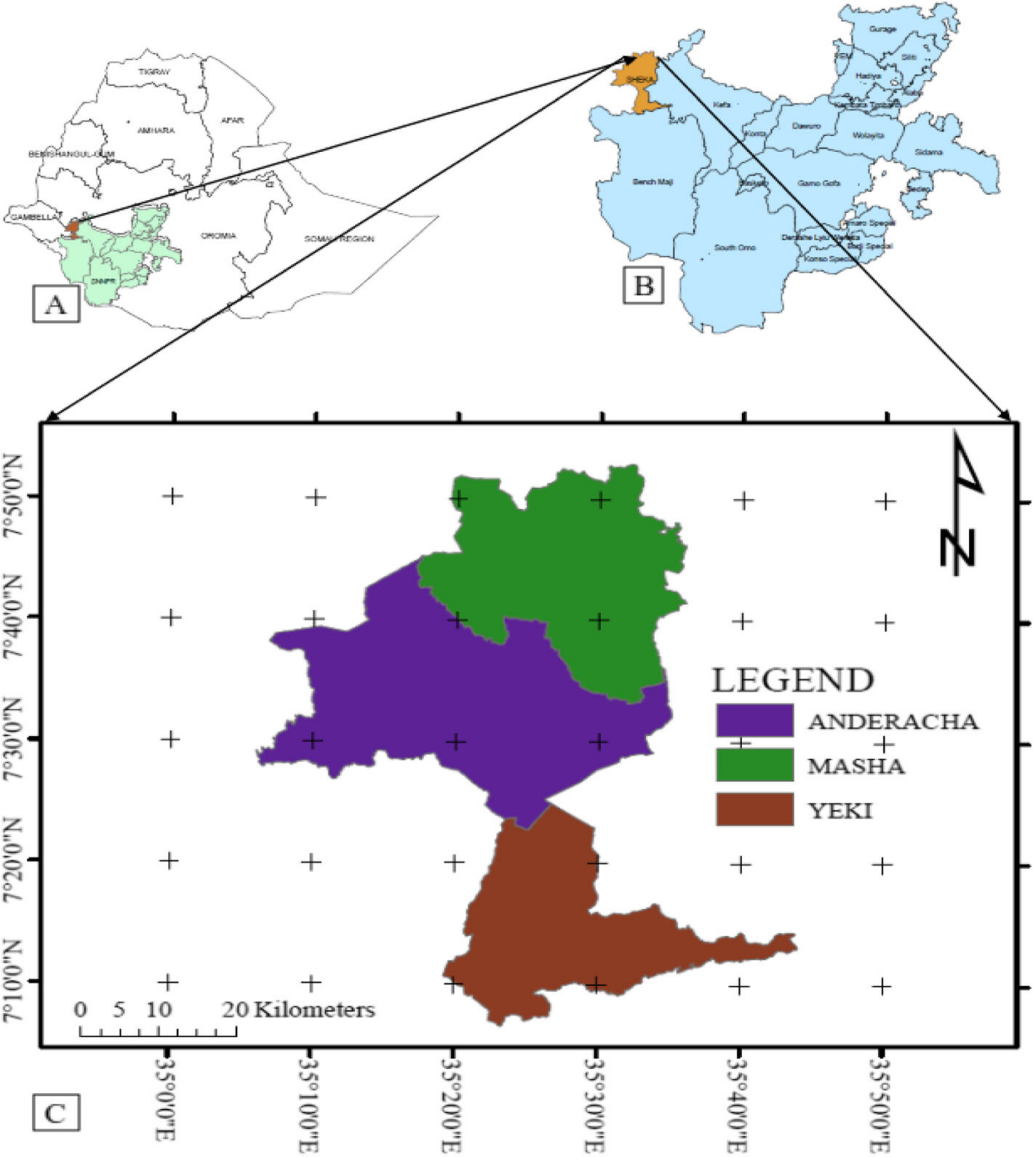
Map of Ethiopia showing the location of the study area. **a** Map of SNNPRS in SW Ethiopia, **b** zones in SNNPRS, and **c** Districts in Sheka Zone (Fig. 1)

The aim of this study was to document traditional medicinal use practices by the people of Sheka in southwestern Ethiopia. Sheka Zone is located at approximately 700 km southwest of Addis Ababa in the Southern Nations Nationalities and Peoples Regional State (SNNPRS), southwestern Ethiopia. The geographical coordinates of the study area lies between 07° 07.494′ to 07° 52.301′ N and 035° 16.576′ to 035° 39.516′ E with altitudinal ranges of 950 to 2780 masl obtained through GPS ground data followed by ArcGIS based mapping (Fig. 1). The area receives high amount of rainfall with average between 1800 to 2200 mm per annum [15]. The authors further noted that areas with an annual rainfall between 700 and 2000 mm or more are marked as the moist evergreen Afromontane forests in the western high lands. Hence, the vegetation of Sheka Zone belongs partly to the Moist Evergreen Afromontane Forest and partly to the Transitional Forest vegetation type.

### Site and informant selection

A total of 39 study KEBELES (13 kebeles per district: Masha = 13, Andracha = 13, and Yeki = 13) were selected based on distance from administrative towns (Masha, Gecha, and Tepi), presence/absence of health facilities especially for collecting medicinal plant information, and other infrastructures; roads and transportation facilities. Informant sampling was followed an even distribution from 12 sites established for sampling. Thirty-two informants were taken per site. Sample size for informants was determined following [16]. Hence, sample size =${n}_o=\frac{(t)^2\ast (p)(q)}{(d)^2}$, where *t* = value for selected alpha level of significance at 0.025 in each tail, α = 1.96 in the normal distribution, (*p*) (*q*) = estimates of variance, *d* = acceptable margin of error for proportion being, and *n*_o_ = sample size to be drawn from population (*N*). Individuals with special ethnobotanical knowledge and focus groups were purposively sampled as key informants. A total of 384 informants were interviewed randomly. About 30 key informants were purposively sampled (384 + 30 = 414 informants total). Average distance in km of respondents from respective health centers and frequency of medicinal plant citations were recorded.

### Data collection

Pre-prepared semi-structured interview method was used to retrieve qualitative and quantitative ethnobotanical data from informants [1, 3, 17]. Demonstration, participant observation, and market surveys were also applied. Ethnobotanical data sheet was prepared ahead of time and semi-structured items were incorporated into it to be used during ethnobotanical information retrieval from both general and key informants during actual field work [1, 3]. Field notes were recorded keeping secrete knowledge (taboos and secretes) of the local people [1]. In order to create confidentiality, necessary ethical clearance was done by briefing to the informants. Bennett’s Golden Rules (being truthful, sharing, considering values and religion, respect, learning from people, intellectual and real property rights, listen to people, ask permissions, respect secrets) for ethnobotany fieldwork [18].

### Market surveys, group discussions

To get general information on the multipurpose roles and marketability of medicinal plant species, a total of 15 market surveys: 12 market surveys from the 12 village centers (one market survey per village center) and three market surveys (one market survey per each town center) from the three town centers were made. Marketable plant species and their plant-derived products sold on markets were recorded. The type of plant species, place of its origin (wild, home garden, far from/near to the market place), processed/unprocessed, plant parts marketable, drivers of marketability (prices, medicinal value, food value), and implications to species rarity/abundance for conservation, management, and sustainable use. Five to ten groups were formed per the 12 village centers and discussions were made twice in two rounds to validate the information retrieved avoiding biases.

### Guided field walk

The methods of guided field walk were applied through negotiation with the respective field guide to each site. Accessible sites with associated risk factors were first identified before starting the actual field walk. Field guides from respective town centers as well as village centers were selected based on their willingness, ability to walk long distances within the forest, general plant knowledge in local language (SHEKINANO), and ability to translate the Shekinano terms into Amharic, English, or Afaan Oromoo (the three languages that the research can easily understand). Guided field walk help to create an opportunity to make note on the habit, habitat, appearance, and the relationships of medicinal plants with other species (plant associations). In the meantime, all possible sensations such as seeing, feeling, smelling, and tasting of the medicinal and wild edible plants under question were made to understand the unique feature of the species. Moreover, traditional healers who helped during the guided field walk also played crucial role in identifying the medicinal and wild edible plants encountered in the field by providing its vernacular names, medicinal use, parts used, preparations, dosage, and traditional applications. Voucher specimen collection by guided field walk was supported by digital photographing of both fresh specimens and pressed dry specimens. The specimen collection was conducted in the wild, home gardens, and markets.

### Ethnobotanical data analysis

Ethnobotanical data were analyzed following the basic analytical tools [1, 19, 20]. Potentially effective medicinal plants were identified by the method of informant consensus factor (Trotter and Logan 1996 in [21]. Hence, $ICF=\frac{n_{ur- nt}}{n_{ur-1}}$, where ICF = informants consensus factor, *n*_*ur*_= relationships between number of each use category of medicinal plants, and *n*_*t*_ = number of taxa used. Simple preference ranking, direct matrix ranking, and paired comparisons were done to test for the consistency in responses, single and multiple dimensions of responses, transitivity, as well as clustering techniques [1]. The numbers of pairs of objects to be compared were computed as: number of pairs of objects (NP) $=\frac{n\left(n-1\right)}{2}$, where *NP*= number of pairs of objects/items to be compared and *n* = number of objects/items to be compared. The relative healing potential of each reported medicinal plant used against human aliment was calculated as fidelity level (FL) computed as: $\mathrm{FL}\;\left(\%\right)=\left(\frac{I_P}{I_U}\right)\;\mathrm{x}\;100$, where, *FL*= fidelity level or relative healing potential, *I*_*P*_ = the number of informants who independently cited the importance of a species for treating a particular diseases (frequency of citation of a species for a particular aliment), and *I*_*U*_ = the total number of informants who reported the medicinal plant for a given diseases (total number of citations of that species) [20]; Frieman et al. 1986 cited in [22].

The Shannon-Wiener use value diversity index for overall use values of the entire species data set was calculated as UVDs $=\sum \limits_{i=1}^S UVi\ln UVi$,

where *S* = is the number of species in the entire data set, UV = a simple sum of all known uses for each species, UVi = the relative use value of species i, and lnUVi = the natural logarithm of the relative use value of species *i* [23].

## Results

### Medicinal plant diversity

**Fig. 2:**
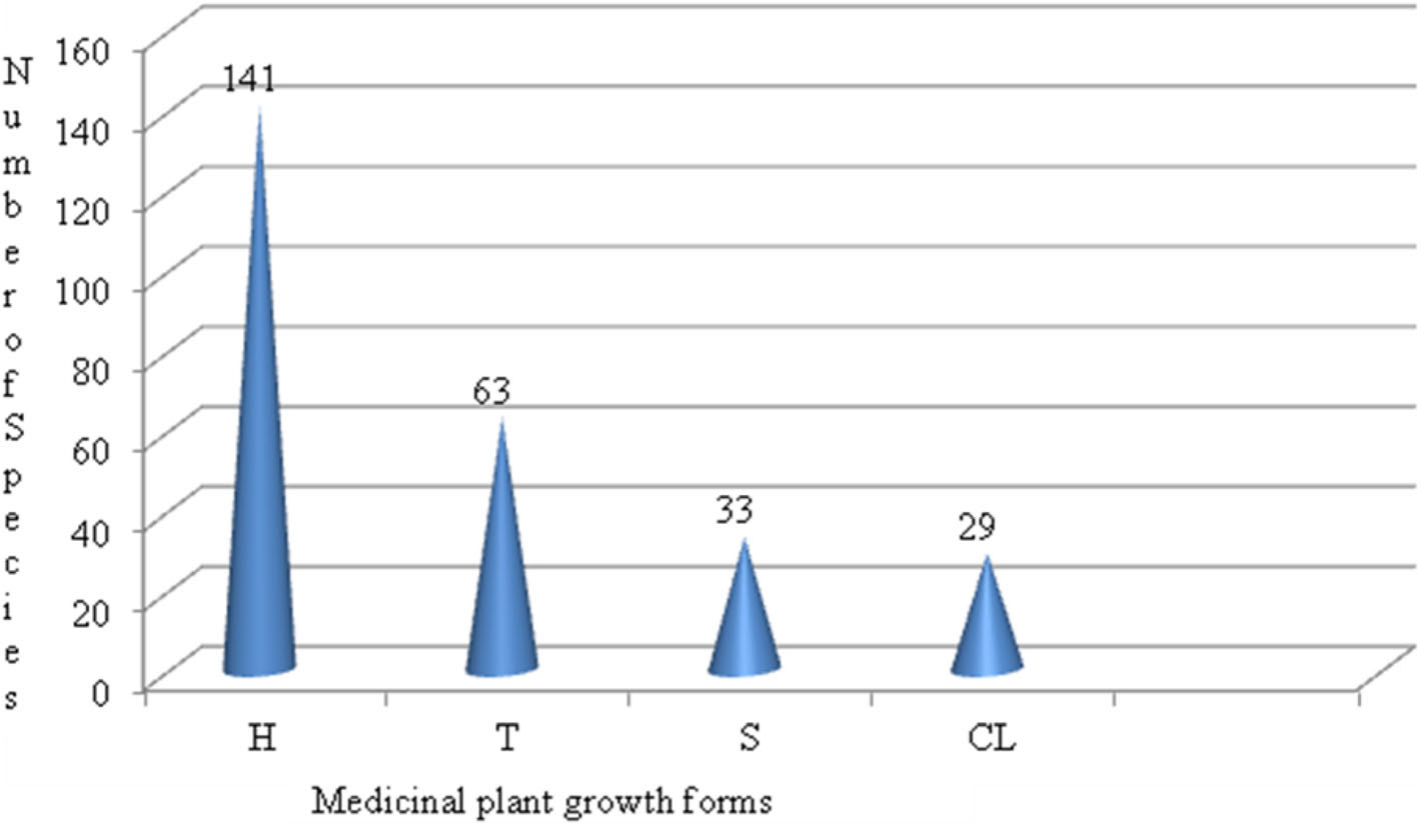
Distribution of medicinal plant growth forms

A total of 266 plant species belonging to 192 Genera and 74 Families were identified to have medicinal value in the study area (Additional file 1). These species were used primarily to treat major health problems of both human and livestock. From the total 266 medicinal plants, 204 (77%) of them were used to treat human health problems; only ten (4%) of them were used to treat livestock health problems and 52 (19%) of them were used to treat both human and livestock health problems. The four major medicinal plant growth forms identified from the study area were herbs, shrubs, climbers, and trees. The result of analysis of diversity of medicinal plant growth forms is indicated in Fig. 2. Letters in the figure refer to H = herbs, T = trees, S = shrubs, and CL = climbers.

### Medicinal plant parts used

**Fig. 3:**
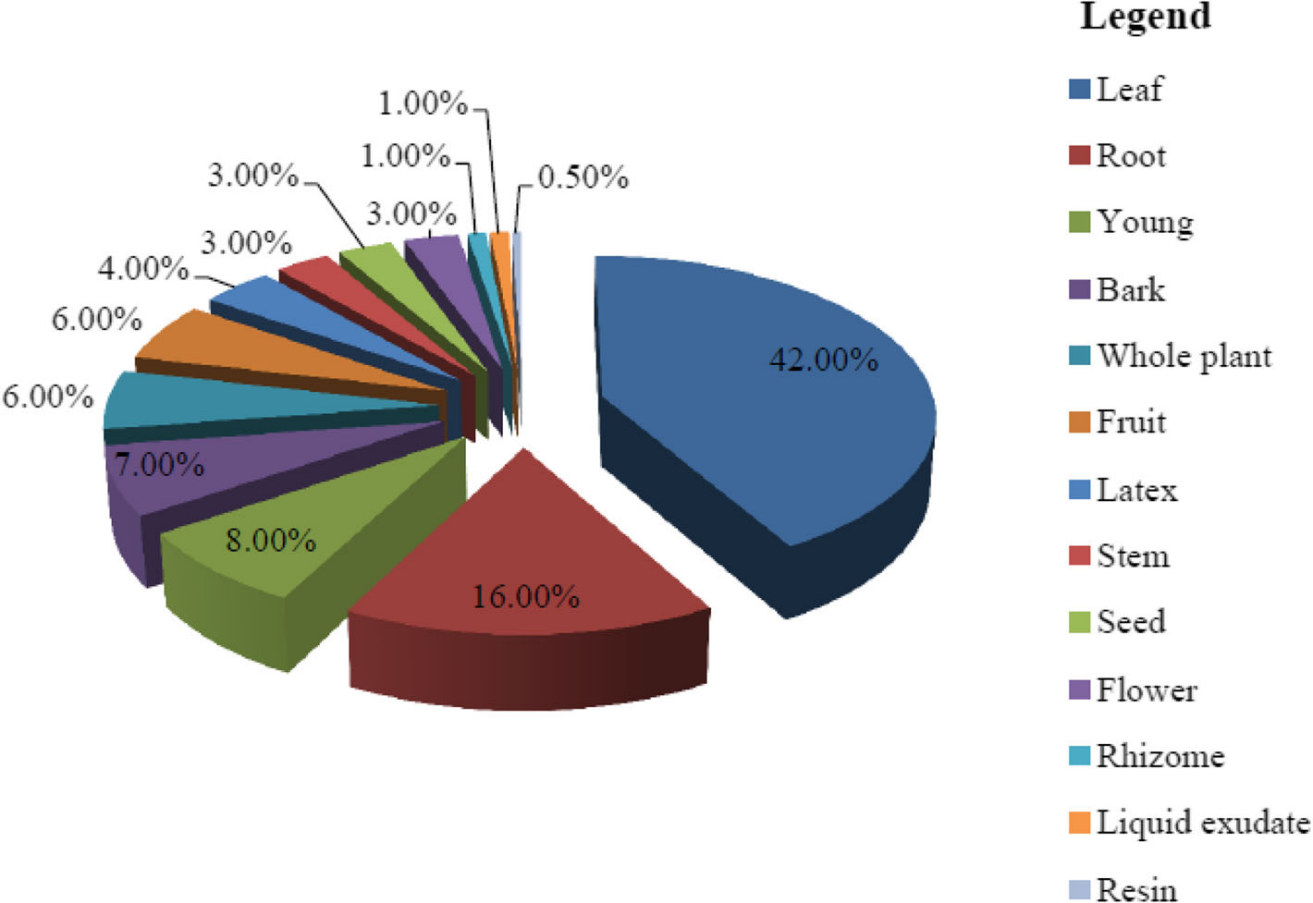
Distribution of medicinal plant parts used in the study area

Results of analysis of medicinal plants used in the study area indicated that 13 medicinal plant parts were identified as major parts used for treating various health problems. These are leaf, (L) (178, 42%), root (R) (66, 16%), young shoot (Sht) (32, 8%), bark (Bk) (28, 7%), whole plant (Wp) (26, 6%), fruit (Fr) (25, 6%), latex (Lx) (18, 4%), stem (St) (14, 3%), seed (Se) (13, 3%), flower (Fl) (11, 6%), rhizome (Rh) (6, 1%), liquid exudate (Lq) (3, 1%), and resins from mature stem (Res) (0.5%) (Fig. 3). Hence, fresh leaf preparation constitutes the largest percentage of plant parts used as medicines.

### Condition of preparation

**Fig. 4:**
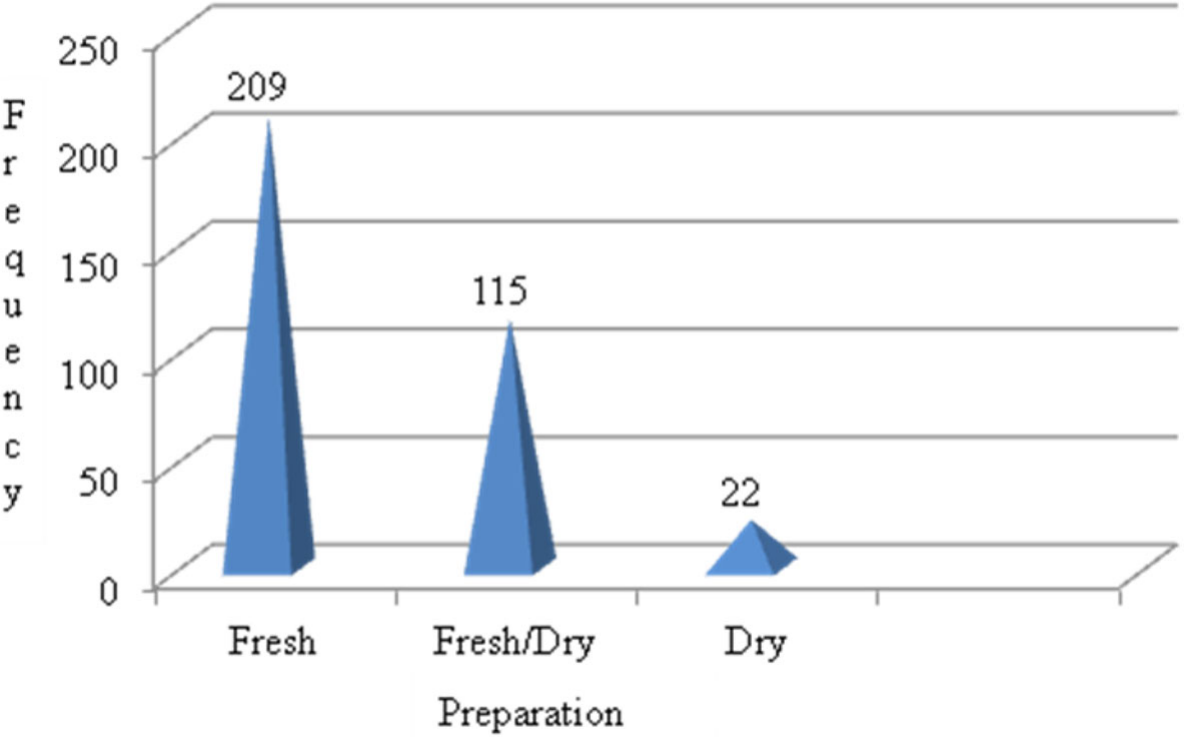
Condition of preparation of medicinal plant parts

Results of analysis for condition of preparation versus total number of citations by informants indicated that out of a total of 346 conditions of preparation reports, the majority of the medicinal plants were shown to be prepared from fresh plant materials only (60.40%) followed by fresh or dry condition (33.24%). Only few medicinal plants were prepared from dry plant material (6.36%) alone (Fig. 4). The above figures indicate that traditional healers claim some medicinal plant parts as showing biological activities only when prepared from fresh materials while others are active if prepared either from fresh or dry plant materials. In others, it was prepared from only dry materials to be stored for long-term use without losing their healing potential.

### Route of administration

**Fig. 5:**
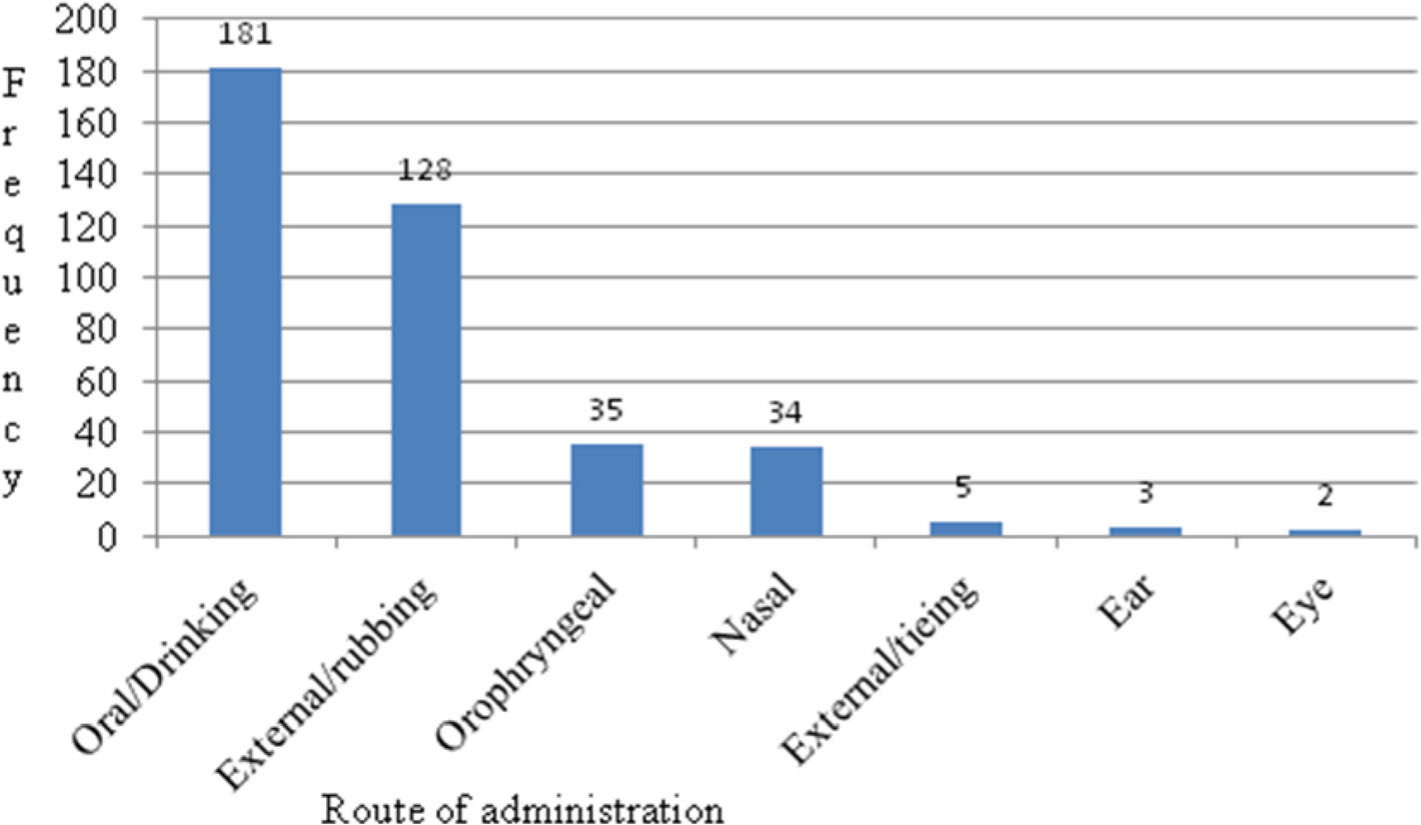
Route of administration of medicinal plants

Results of analysis of route of administration of medicinal plants revealed that the medicinal plants were administered through oral/drinking (Orl) (47%), external/rubbing (Ex/R) (33%), oropharyngeal (Orgl) (9%), external/sealing (Ex/Seal) (1%), nasal (Na) (9%), ear (Er) (1%), and eye (Ey) (1%) (Fig. 5). The use of terms for route of administration of medicinal plants in the context of this article was: Oral means the medicinal plant is taken orally in the form of liquid drink or solid material into stomach; External/rubbing means the medicinal plant is applied to the external part of the body in the form of liquid ointment usually to the skin; Oropharyngeal means the medicinal plant is applied to the mouth and the pharynx usually against gingivitis, tonsillitis, and toothache; External/sealing means the medicinal plant preparation is used to treat wound on the body by tightly tying on the affected part of the skin; Nasal means the medicinal plant is taken through the nostrils in the form of sniff or used as an ointment around the nose cavity; Ear means the medicinal plant is applied in the form of ear drops in liquid form; and Eye means the medicinal plant is applied in the form of eye drops in liquid form or chewed and spited into the eye in solid form.

### Common health problems in the study are and informant consensus factor

The major types of health problems identified from the study area were categorized into 22 types of major disease categories locally treated with medicinal plants in the study area. The traditional versus clinical explanations of these health problems were indicated in Table 1.

**Table 1:** List of top 20 most cited human health problems in the study area

Shekinano terms	Clinical terms	Clinical explanations [24–26]
Mache bewo/	^a^Stomachache	Any problems related to stomach either due to parasites, infections or allergy
Michatto	Allergy	Hypersensitive of the body to particular antigens
T'inbate	Jaundice	A yellow discoloration of the skin or whites of the eyes
Mawo/mac'e/mae'	wound	Any infectious or mechanical injury to part of the body either with pus or dry
Wasfato	Ascariasis	A disease caused by infestation of *Ascaris lumbricoides*
O'tio/Shouka o'ttio	common cold	A widespread infectious virus disease causing inflammation of mucus membrane
Kette bewo	Tonsilitis	Inflammation of the tonsils due to bacteria or viral infection causing sore throat
Machichoro	Endoparasites	Parasites living in the inside of their hosts
Eange kajjo	Headache	Any disorder related to mental malfunctioning leading to loss of self-personality
Shulit kunane shac'o	Rabies	An acute virus disease of the central nervous system of all warm blooded animals
Shikekisso	Fungal	Any health problem resulting from fungal attack or infection
Bic'o	Bloody diarrhea	Diarrhea with bloody discharge
K'op'aro	Cockroaches	A large brown insect with wings living in houses especially in dirt
Dingare atto	Snake poison	Related to any species of snake that is dangerous to humans. It is general term
Afee shurite	Eye disease	Bloodshot, watery, dry and itchy eyes/painful spot or acne on the eyelids
Maac'ec'ot't'o	Parasites	Any living thing that lives in another living organism
Gatach bewo	Amoebiasis	An infection of the intestinal tract causing severe bloody diarrhea
Digare/T'ekare	Snake bite	Wound resulting from snake bite through which the snake injected into the victim
Chogare bewo	Gastritis	Inflammation of the lining of the stomach either acute or chronic stage
Gocho/Goche bewo/	Diarrhea	Frequent bowel evacuation or the passage of abnormally soft, liquid faeces

^a^Non-parasitic/non-specified stomach problem. The 20 most cited is out of *n* = 143 health problems cited by informants at zone level

The informant consensus factor is used as a parameter to rank human health problems. Beforehand, secondary data about the existing health problems in the entire zone were collected from health institutions (zone and district health divisions). Then these data were compared with the data collected during the actual field work. Finally, the whole health problems were categorized based on whether they are infectious or non-infectious diseases, deficiency or non-deficiency diseases, systemic or non-systemic, respiratory or non-respiratory, circulatory or non-circulatory, and the like.

The informant consensus factor (ICF) values of the 22 different categories of human diseases are shown in Table 2.

**Table 2:** Informant consensus factor values of top 22 human health problem categories

a	Diseases/categories	nt	nur	nur-nut	nur-1	ICF
1	Dermal/skin diseases/	16	399	383	398	0.96
2	Poisoning/snake, insect, other/	19	317	298	316	0.94
3	Gastrointestinal	23	293	270	292	0.92
4	Allergy	35	285	250	284	0.88
5	Viral/Rabies	14	92	78	91	0.86
6	Fungal	32	149	117	148	0.79
7	Ecto-parasites	27	105	78	104	0.75
8	Headache	23	87	64	86	0.74
9	Oropharyngeal	58	179	121	178	0.68
10	Respiratory	50	147	97	146	0.66
11	Cardiac/systemic/	47	123	76	122	0.62
12	Hepatitis/Liver/	65	158	93	157	0.59
13	Opthalmia/eye/	61	143	82	142	0.58
14	Musculoskeletal	12	27	15	26	0.58
15	Renal/Kidney problems/	11	25	14	24	0.58
16	Reproductive	21	46	25	45	0.56
17	Mammary/breast diseases/	10	17	7	16	0.44
18	Otitis/ear/	14	21	7	20	0.35
19	Acute fibril illness/nerve/	9	13	4	12	0.33
20	Night evil	3	4	1	3	0.33
21	Glandular fever	6	8	2	7	0.29
22	Epilepsy	4	5	1	4	0.25

*nt* number of medicinal plant species (taxa used), *nur* number of use citations

^a^Indicates 22 major categories of *n* = 143

### Relative healing potential of medicinal plants, fidelity level

The relative healing potential or fidelity levels of top 22 medicinal plants against major human health problems are given in Table 3.

**Table 3:** Fidelity level of medicinal plants against a given human aliment

SN	Scientific names	Therapeutic category	^a^Ip	^a^Iu	%FL
Fidelity level of most cited medicinal plants against a given human aliment (*N* = 204)					
1	*Solanecio mannii* (Hook.f.) C.Jeffrey	Jaundice/T'INBATO/	15	18	83.33
2	*Hagenia abyssinica* (Brace) J.F.Gmel.	Tapeworm	17	28	60.71
3	*Rumex abyssinicus* Jacq.	Jaundice/T'INBATO/	16	27	59.26
4	*Cucurbita pepo* L.	Ascariasis	15	31	48.39
5	*Eragrostis tef* (Zucc.) Trotter	Used as antidote	18	38	47.37
6	*Nigella sativa* L.	Asthma	19	43	44.19
7	*Leucas jamesii* Bak.	Canker sore	15	34	44.12
8	*Cynoglossum amplifolium* Hochst. ex A.DC. inDC.	Allergy/MICHATO/	25	67	37.31
9	*Cynoglossum lanceolatum* Forssk.	Allergy/MICHATO/	25	67	37.31
10	*Ocimum urticifolium* Roth	Endo-parasites	20	70	28.57
11	*Cynoglossum coeruleum* Hochst. ex A.DC. inDC.	Allergy/MICHATO/	30	115	26.09
12	*Euphorbia ampliphylla* Pax	Ascariasis	18	76	23.68
13	*Pycnostachys eminii* Gürke	Allergy/MICHATO/	19	87	21.84
14	*Pycnostachys meyeri* Gürke	Allergy/MICHATO/	19	87	21.84
15	*Vangueria madagascariensis* Gmel.	Endo-parasites	27	124	21.77
16	*Dombeya torrida* (J.F. Gmel.) P. Bamps	Jaundice	15	85	17.65
17	*Peperomia retusa* (L.f.) A.Dietr.	Stomachache	35	217	16.13
18	*Ruta chalepensis* L.	Evil eye	21	137	15.33
19	*Phytolacca dodecandra* L Ήѐrit.	Rabies	24	157	15.29
20	*Ocimum lamiifolium* Hochst. ex Benth.	Parasites	23	181	12.71
21	*Momordica foetida* Schumach.	wound	21	201	10.45
22	*Prunus africana* (Hook.f.) Kalkm.	Jaundice/T'INBATO/	19	275	6.91

^a^I = refers to all informants both key informants and non-key informants (*n* = 414). Ip = the number of informants who independently cited the importance of a species for treating a particular diseases, Iu = the total number of informants who reported the medicinal plant for any given diseases, FL = fidelity level (relative healing potential)

The therapeutic categories (TC) of the health problems indicated in the tables are summarized based on the general information retrieved from the informants. The therapeutic categories were latter crosschecked with the health professionals at Sheka Zone Health Division and district health centers for correctness of medical and clinical terms (Additional file 3). Note that some health problems are restricted to only humans while others are restricted to only livestock. Those health problems reported as common to both humans and livestock are zoonotic (communicable from livestock to livestock).

The health problems identified include both infectious diseases (bacterial, viral, fungal, protozoan, gastrointestinal parasites) and non-infectious diseases (mechanical injuries, allergic reactions, deficiency diseases, nervous, and psychomotor disorders). The 20 most cited diseases according to the ethnomedicinal information retrieved from the informants include stomach problems (gastrointestinal problems), allergy, jaundice, wound, ascariasis, common cold, tonsillitis, endo-parasites, headache (mental problems), rabies, fungal infection, bloody diarrhea, cockroach borne diseases, snake poisoning (non-bite), snake poisoning (bite), eye diseases, parasites, amoebic dysentery, gastritis, and non-bloody diarrhea.

### Paired comparison

Results of test for consistency and transitivity through paired comparison for top five medicinal plants against gastrointestinal problems was obtained by simple preference ranking. Results of paired comparison of the five medicinal plants against gastrointestinal problems as obtained from ten respondents (R1-R10) are indicated in Table 4.

**Table 4:** Results of paired comparison of five medicinal plants against gastrointestinal problems

Medicinal plants	Respondents (*R*)											
	1	2	3	4	5	6	7	8	9	10	Total	Rank
*Celosia schweinfurthiana*	0	2	1	0	3	0	1	0	0	1	8	5th
*Croton macrostachyus*	4	3	4	3	2	3	4	3	3	4	33	1st
*Lobelia giberroa*	1	1	0	2	4	2	0	1	2	0	13	4th
*Peperomia retusa*	2	0	3	4	0	1	2	2	1	2	17	3rd
*Prunus africana*	3	4	2	1	1	4	3	3	4	3	28	2nd

#### Knowledge differences according to age, gender and literacy level of informants

Summary of statistical test of significance on the average number of medicinal plants cited among different informant groups in Sheka Zone is indicated in Table 5. The results of the analysis shows knowledge differences among different informant groups based on the specified parameters as indicated in the table.

**Table 5:** Comparison of knowledge differences among different groups of informants

Parameters used	Informant groups	n	Average ± SD	*Z* value**	*P* value*
Gender	Male	380	5.542 ± 3.7248	*z* = − 1.9908	0.0465
	Female	34	4.765 ± 1.9857		
Age	18–30 (youngers)	87	4.149 ± 0.2178	*z* = − 5.5160	0.00001
	> (Elders)	327	5.832 ± 0.2135		
Literacy level	Illiterate	241	6.295 ± 0.2112	*z* = 5.4882	0.00001
	Literate	173	4.341 ± 0.2865		
Informant category	Key informants	30	13.367 ± 1.4259	z = − 5.9481	0.00001
	General informants	384	4.862 ± 0.1051		
Total number of informants (*N*)		414	–	–	–

*Significant difference (*p* < 0.05); **z (0.05) (two-tailed), df = 412, *N* = 414, *n* = number of respondents

### Use diversities of medicinal plant species

The Shannon-Wiener use value diversity index for overall use values of the entire species data set with all specific uses recorded for 14 use categories was 5.155. The total number of medicinal plant species in the entire data set was 266. The use diversity values for the upper 20 individual plant species are indicated in Table 6. These species can be further considered high ranking medicinal plants based on their use value diversities.

**Table 6:** Use diversity indices of 20 high ranking medicinal plant species in the entire data set

Species	Family	^a^UVs	UVi	lnUVi	Abs(lnUVi)	UVilnUVi
*Syzygium guineense subsp. afromontana*	Myrtaceae	43	0.020	− 3.937	3.937	0.077
*Syzygium guineense subsp.marocarpa*	Myrtaceae	43	0.020	− 3.937	3.937	0.077
*Croton macrostachyus*	Euphorbiaceae	41	0.019	− 3.985	3.985	0.074
*Manilkara butugi*	Sapotaceae	37	0.017	− 4.088	4.088	0.069
*Ekebergia capensis*	Meliaceae	34	0.015	− 4.172	4.172	0.064
*Ilex mitis*	Aquifoliaceae	34	0.015	− 4.172	4.172	0.064
*Olea welwitschii*	Oleaceae	34	0.015	− 4.172	4.172	0.064
*Arundinaria alpina*	Poaceae	33	0.015	− 4.202	4.202	0.063
*Prunus africana*	Rosaceae	33	0.015	− 4.202	4.202	0.063
*Hallea rubrostipulata*	Rubiaceae	30	0.014	− 4.297	4.297	0.058
*Millettia ferruginea*	Fabaceae	30	0.014	− 4.297	4.297	0.058
*Schefflera abyssinica*	Araliaceae	30	0.014	− 4.297	4.297	0.058
*Cordia africana*	Boraginaceae	29	0.013	− 4.331	4.331	0.057
*Schefflera volkensii*	Araliaceae	29	0.013	− 4.331	4.331	0.057
*Hagenia abyssinica*	Rosaceae	27	0.012	− 4.403	4.403	0.054
*Milicia excelsa*	Moraceae	27	0.012	− 4.403	4.403	0.054
*Ozoroa insignis*	Anacardiaceae	26	0.012	− 4.440	4.440	0.052
*Ozoroa pulcherrima*	Anacardiaceae	26	0.012	− 4.440	4.440	0.052
*Podocarpus falcatus*	Podocarpaceae	26	0.012	− 4.440	4.440	0.052
*Trichillia dregeana*	Meliaceae	26	0.012	− 4.440	4.440	0.052
$$ \mathrm{UVDs}=\sum \limits_{i=1}^S UVi\ln UVi=5.155 $$						

*UVDs* use value diversity, *UVs* a simple sum of all known use for each species, *UVi* the relative use value of species i, *lnUVi* the natural logarithm of the relative use value of species *i*, *Abs*(*lnUVi*) the absolute value of lnUVi, *S* the total number of medicinal plant species in the entire dataset

^a^Only species with total use value of all known use categories > 26 are indicated (*S* = 266, use categories = 14)

## Discussion

### Medicinal plants

The vegetation of Sheka Zone is rich in medicinal plant diversity and floristic composition. A total of 266 (48%) of the total plant species recorded from the area were found to have medicinal values in one or more ways either directly or indirectly indicating that the vegetation of Sheka is good reservoir of plant species of medicinal values. According to [27], high diversity of medicinal plants is attributed to good vegetation cover which in turn implies their significant role in plant-based traditional medicine in meeting basic primary healthcare needs. Comparing to the previous studies, the current study reported relatively high number of medicinal plant species. For instance, it was reported that [28], 173 species [29];, 135 species [12];, 91 species [30];, 27 species [27];, 71 species [31];, 35 species [32];, 51 species [33];, 120 species [34];, 230 species [13];, 83 species [35];, 67 species from different parts of Ethiopia.

These medicinal plants are distributed among forests, home gardens, river basins, and stream sides, road sides, along valleys, wetlands, farmlands, and coffee and tea cultivations, epiphytic on large tree trunks. Similar studies on Ethiopian medicinal plants also showed that traditional medicinal plants are harvested mainly from wild habitats than home gardens [30, 31, 34]. Traditional healers know the location of these valuable medicinal plant species and through intense guided field walk they can directly locate them. With great care and patience, it becomes the task of the researcher to retrieve unbiased ethnobotanical information from the local healers keeping the top secrecy of their cultural beliefs and medicinal plant knowledge.

The medicinal plant species recorded from Sheka Zone were used to treat humans 204 (77%), livestock 10 (4%), and both humans and livestock 52 (19%) health problems. The 13 plant parts were identified as parts used to treat about 143 health problems which further categorized into 22 types of diseases locally treated by traditional healers. The most frequently cited medicinal plants such as *Croton macrostachyus*, *Prunus africana*, *Rumex nepalensis*, *Justicia schimperiana*, *Achyranthes aspera*, and many others are also reported by many researchers conducted in other parts of Ethiopia. For instance, 25 of the medicinal plants used to treat human ailments and eight of the medicinal plants used to treat both human and livestock ailments recorded in the current study are also reported by [36]. Similarly, 29 of the medicinal plants recorded as threating human ailments in the current study are also reported by [37]. Moreover, Solanaceae, Asteraceae, Lamiaceae, Fabaceae, and Euphorbiaceae are medicinal plant families with the highest number of plant species used in the treatment of human and livestock ailments in Erobe and Gulomeheda Districts of Tigray [38].

#### Diversity of medicinal plant growth forms and parts used as medicine

The vast majority of the medicinal plants 141 (53%) collected from the study area belonged to herbaceous species. They account for more than 50% of the total medicinal plants. They were major reservoirs of medicines for local people of Sheka Zone. High usage of herbs as sources of herbal remedies might attribute to their relative abundance and ease of accessibility to healers. Study conducted by [32, 39] in southwestern Ethiopia reported that high usage of herbaceous medicinal plants was attributed to their relative abundance as compared to other plant growth forms and history of settlement the people using it. Moreover, the patterns of growth could also contribute the high frequency of usage of herbaceous plant species due to the fact that herbs are the dominant plant growth forms in the Ethiopian flora.

Large numbers of medicinal plants in the study area were also found to be used to treat only human health problems and only few of them were reported to be used to treat livestock ailments. The possible reasons could be attributed to the relative preference to and emphasis of the people on human health problems as compared to livestock health problems. Moreover, relatively larger number of medicinal plants were used for treating both human and livestock ailments. Availability of veterinary clinics could also be a factor as reported by [39] because people prefer modern healthcare services for their livestock in the presence of such services in their vicinity.

#### Plant parts used as source of medicines and implications

The study indicated that the most frequently used plant parts are leaves (66.93%) and roots (24.81%). It agrees with other ethnomedicinal studies in Ethiopia that showed leaves as the most frequently used plant parts [13, 14, 27, 28, 30, 33] followed by roots [12, 32, 34]. Some plant parts particularly the root, leaf, and bark are sensitive to harvesting so that affecting them could directly or indirectly affect the life of the whole plant. This is due to the fact that these plant parts play vital role in the whole life cycle of the plant species under question. Rare species for instance may be susceptible to local extinction due to over usage and pressure posed on its sensitive organs if care is not taken. Typical example is the case of *Echinops kebericho* whose root was highly marketable in local markets of Sheka Zone. It is obvious that uprooting the species could kill the individual plant leading to reducing its availability in its natural populations. Studies elsewhere in Ethiopia also indicated that over usage is a threat posing pressure on plant species in general and medicinal plants in particular [37]. Medicinal plant parts used could also serve as target organs for further medicinal plant profiling, promoting, and drug development.

#### Preparation and application of medicinal plants

In the current study, greater than 60% of the medicinal plant preparations were fresh plant material (Additional file 2). Similar finding was reported by [14, 27–30, 33] that the fresh plant material is the most commonly used condition of preparation. Traditional healers claim that some medicinal plants lose their healing potential if not used in fresh condition. The implication was that there was limited practice of dry storage for future use. It means that there could be increasing frequency of harvesting which may affect the medicinal plant in use or its parts. Similar studies also confirmed that freshly harvested medicinal plant parts were frequently used in the preparation of plant derived remedies [39, 40].

#### Route of administration of medicinal plants and implications

According to the current study, the majority of the routes of administrations of the medicinal plants were internal through oral intake. However, there is no guarantee about the side effects of such type of medicinal plant intake. There may be high chance of health complications to arise creating both short term and long term problems on the life of the patient. Giday [39], for instance, reported that relatively less risk of being poisoned by improper use of herbal remedies was external/skin application as compared to internal/oral applications. The implication was the presence of problems of dosage, standardization, side effects, validity, and the susceptibility of delicate body parts of the patient above all. Hence, there is a need to give priority attention to the establishment of standardized traditional treatment guidelines for medicinal plants by well-known traditional healers. Ethnomedicinal studies such as [14, 29, 35, 40, 41] reported that oral administration is the most commonly used route followed by external/skin creaming.

#### Dosage determination of medicinal plants and implications

Traditionally, healers use different methods as means of dosage determination. Among these were finger strips of little finger, finger nails of little finger, glass, coffee cup, and teaspoon based on the age and sex of the patient. This study is in line with the reports of [29, 42] that dosage varies according to age, sex, and physical condition of patients. Moreover, they use different preparations (mixed plant extracts), milk, honey, meat soup, bread of red teff as antidote against the side effects. However, there is a high chance of the patient to be victim of the side effects of the medicinal plant in use and it is obvious that the scenario is even true in modern medical care services if great care is not taken. For that matter, the Food, Medicine, and Health Care Administration and Control Authority (EFMHACA) of Ethiopia, for instance, has already established standard treatment guidelines at various levels of health facilities (health centers, primary hospitals, and general hospitals) [24] for multi-stage treatment services. Yet the traditional treatment practices have a number of gaps in it despite its vital role in primary healthcare services.

#### The informant consensus factor values and its implications

For the current study, strong agreement among informants (greater than 50%) was observed for 16 of the 22 human health problems. The informant consensus factor (ICF) value for epilepsy was only 25% for the current study. Informant consensus values normally range between 0 and 1 [43]. High informant consensus factor values were observed for treating both human and livestock ailments in the study area. The implication was that only few medicinal plant species were reportedly used by very high proportion of informants to threat a given category of health problems. That means there was strong agreement among informants over which medicinal plant to use a in the traditional treatment of a given health problem. Low ICF values show informants’ disagreement over which medicinal plant species to use for treating a given category of health problem. It was stated that ICF is used to identify plants of particular intercultural relevance [43]. Hence, it would be necessary to group health problems into wide diseases categories.

#### Interpretation of the values of informant consensus factor

The ICF values for the 22 major human diseases categories range in between 0.25 and 0.96 with average value of 0.62 as indicated in Table 2 of the results section. Since the values of informant consensus factor normally ranges between 0.00 at its lowest and 1.00 at its highest [23, 43], it implies that there is strong agreement among informants (> 50%) for the 16 of the health problems. High informant consensus factor values imply strong agreement of informants on which medicinal plant to use to cure specific type of aliment. Low informant consensus factor values on the other hand imply strong disagreement of informants on which medicinal plant to use to cure specific diseases. It means that if ICF value is high, few medicinal plants species are reported to be used by high number of informants to threat a particular category of health problem and vice versa. Hence, more than 90% of informant consensus factor was obtained for skin diseases (96%), poisoning/snake, insect bite (94%), and gastrointestinal (92%).

Parasitic infections such as scabies, pediculosis, and onchocerciasis were the commonest health complaints followed by bacterial and fungal infections in southwestern Ethiopia [44]. However, care should be taken while using such outdated literature sources and up-to-date research findings about the current status of various health problems should be referred. Onchocerciasis, for instance, was almost under control in Ethiopia and no significant case reports are available in the current situation. Recent research findings show that more than 40% of tropical health problems including malaria were caused by gastrointestinal parasites in developing countries [45, 46]. In Ethiopia, common helminthic infections for which traditional remedies were highly prescribed include tapeworm, ascariasis, hookworm, and pinworms [47]. A study conducted around Tepi Town of Sheka Zone also revealed that *Ascariasis* and *Trichuris trichiura* were the most common helminthes in the area [48].

#### Fidelity level of medicinal plants and its implications

The fidelity level of medicinal plants represents the relative healing potential of medicinal plants against a given ailment. In the current study, the relative healing potential or fidelity level (FL) of most sited medicinal plants with relatively higher fidelity level values for treating human, livestock, as well as both human and livestock aliments were identified and discussed. Relatively high fidelity levels were observed for medicinal plants the medicinal plant species have relatively high healing potential against the respective health problems mentioned. In other words, plants with high FL values could be target species prioritized for conservation, management, and sustainable use after their bioactivities were properly evaluated and confirmed. They could also contribute to medicinal plant data base. It was reported that lower fidelity level indicates a given medicinal plant species could have more number of mentions by the informants than medicinal plant species that have high fidelity level [43].

Considerable number of medicinal plants in Sheka Zone need further chemical profiling to assure their validity and efficacy. According to Heinrich [21], he noted that systematic evaluation of indigenous therapeutic methods and practices so as to improve healthcare in marginalized regions became an important element of the agenda of international and national organizations. Validation of therapeutic claims helps to increase confidence and generate income creating opportunity for marketing of herbal medicine [49].

The relative importance of a given medicinal plant within a culture in which it is found to be significant is evaluated through the application of quantitative ethnobotanical methods and data comparisons among diverse cultural groups within a given fragment of social groups or community. Quantitative ethnobotanical methods and approaches such as the use of informant consensus factor, relative healing potential, relative cultural importance, cultural significance index, ranking, and scoring are among the indices used in the systematic evaluation of the medicinal plant in need. Moreover, use variability of medicinal plants of interest in search of their bioactive compounds can be estimated by using the informant consensus factor (ICF) values. Hence, plants with the greatest bioactivity are considered to have the highest ICF values and are better candidates for bioprospecting and further profiling [50, 51]. Hence, the considerable number of medicinal plants recorded from Sheka Zone need further profiling to assure their validity and efficacy. As reported by [12], validation of bioactivity of medicinal plants preferred by traditional healers increase their acceptance both nationally and internationally for healthcare systems. Moreover, the findings of [27, 32] summarized that priority for further pharmacological studies must be given to medicinal plants scoring the highest fidelity level.

#### Major health problems in Sheka Zone/Emic versus etic perspectives

The etic/emic approach helps to visualize the way local people try to perceive their surroundings thereby seeking solutions to major practical problems in health, food security, social integrity, and environmental sustainability. Modern science has much to learn from traditional practices as the traditional practice has to learn a lot from modern science in all aspects of life related to the issues outlined above. For instance, it is a known fact that long before the discovery of modern healthcare systems and drugs, ancient people traditionally used to get self-medication by trial and error. Such traditional therapy was what traditional healers of today still engaging in, although the way they are doing it is closer to modernity. Hence, knowledge integration becomes among important aspects for the success of science. Social beliefs and taboos associated with diseases or any health problems and the associated herbal remedies used to treat such diseases have something to do with the mutual relationships between traditional healthcare system and modern healthcare services. However, relying on traditional healthcare system has yet its own advantages and disadvantages.

On the one hand, traditional healthcare system is believed to be very cost effective, easily accessible, and highly trusted by the patients who get the services if it is carefully performed by well experienced traditional healers. Just as a medical doctor treats his/her patients psychologically well in addition to other medical services, both the traditional healers and the patients in Sheka who are going to get traditional medication have a common belief that God has created the natural medicine, the herbs, and shared his medical knowledge to the authorized person, the traditional healer, so that they confidentially visit the herbalist in their locality to get medication. The healers also believe that God does not refuse them to care for their patients when they give the medicine on behalf of him. Such well-gifted people in Sheka are usually nominated as clan leaders and have specially recognized places in all social aspects in the culture and believe of the Sheka people. They even participate in governance, conflict resolution, and related issues in their society.

On the other hand, there is no evidence about the dosage determination, route of administration of medicinal plants, and the associated short term as well as long term side effects, although traditional healers in Sheka are well-adopted in treating patients. Hence, the issues of validity, standardization, and side effects are questionable so that there is a risk of committing life-threatening events. Even it is well obvious that in well-tested and confirmed modern medical services, there are events where life-threatening cases may occur. These events are related to dosage, patient’s health history, improper prescription of medicines, and related mistakes during multistage treatment options.

The Food, Medicine, and Health Care Administration and Control Authority of Ethiopia for instance prepared standard treatment guidelines for health institutions at various levels [24] which can serve as a standard reference for health professionals. EFMHACA further noted that irrational use of drugs has been one of the major problems in the Ethiopian healthcare system for a long time. It was emphasized that medicines should only be prescribed when necessary, and the benefit-risk ratio of administering the medicine should always be considered prior to prescribing where the prescription should be through the well understanding between the prescriber, the pharmacist, and the patient [24, 25, 52]. The above scenario calls for the need for integrating traditional healthcare system with modern medical services thereby validating, standardizing, and certifying traditional medication and the knowledgeable persons who are giving the service to the society.

#### Knowledge differences according to age, gender and literacy level of informants

From the total of 414 informants, highest numbers of informants (380) were males, whereas only few of them (34) were females due to cultural preseasons. Obviously, ethnobotanical field work is affected by various factors such as cultural background of the society, field situations, willingness of informants, and related sociocultural limitations. Hence, less number of female informants as compared to male informants was interviewed during the current study. A study conducted in Burkina Faso, for instance, showed that it was impossible to interview equal number of men and women due to the traditional rules governing many societies [52]. Age wise, the age range for the entire study was 18 years to 96 years of age. On average, more medicinal plants were reported by male informants (5.542 ± 3.725) than female informants (4.765 ± 1.986) with significance difference (*p* = 0.0465); elders of age > 30 years (5.832 ± 0.213) than youngers of age 18–30 years (4.149 ± 0.218) with significant difference (*p* = 0.00001); illiterate informants (6.295 ± 0.211) than literate informants (4.341 ± 0.287) with significant difference (*p* = 0.00001); key informants (13.367 ± 1.426) than general informants (4.862 ± 0.105) with significant difference (*p* = 0.00001). This study agrees with [31, 53] that older people cited more medicinal plant species than younger people. Moreover, it is in line with [28, 31] that reported illiterate people and key informants are more knowledgeable about medicinal plants as compared to literate people and general informants (Table 5).

#### High ranking medicinal plants

High ranking medicinal plant species are priority species for further profiling against gastrointestinal problem efficacy and safety. Quantitative analytical tools such as ranking and scoring are among the quantitative ethnobotanical approaches used to generate scientifically rigorous results [1, 3]. The authors further noted that pairwise matrix of medicinal plants in relation to a given aliment selected based on the results of ranking and scoring is used to test for the consistency of the relationships of preferences as well as transitivity of results.

Furthermore, pairwise comparison of top five medicinal plants against gastrointestinal problems as obtained from the ten respondents (*R*_1_ through *R*_10_) also show that *Croton macrostachyus* Del. ranked first followed by *Prunus africana* (Hook.f.) Kalkm. *Peperomia retusa* (L.f.) A. Dietr, *Lobelia giberroa* Hamsl, and *Celosia schweinfurthiana* Schinz respectively in this order confirming consistency of relationships and transitivity of results. It implies that the above plant species were found to be culturally important in the study area due to their wide use by a large number of users of the plants due to their curative properties.

Harvesting impacts on multipurpose plant species can be tested by ranking and scoring [1, 5]. It is obvious that there are instances where the most utilized species is going to be most threatened one in its locality if appropriate conservation, management, and sustainable use measures are not taken. This is clear from the point of view of whether the rate at which the species is utilized in the area is much greater than the rate at which it is replacing itself or not in its natural habitats [54]. The worst problem arises when such events are so latent that even it is going to be difficult to take immediate conservation measures to save the rare species. Even species which are not multipurpose but known for their single use value such as medicinal purpose may be at risk of extinction under such circumstances. For instance, medicinal plant species such as *Echinops kebericho* and *Vangueria madagascariensis* were found to be highly wanted species in Sheka Zone for their high medicinal value but they were found to be very rare in their occurrences and distributions in the area and hence they are typical examples.

The route of administration and dosage of medicinal plant plants is usually based on haphazard applications except for few well experienced and knowledgeable traditional healers. Even well-qualified healers are not perfect. The implication of such scenario is that improper use of the medicinal plants can have both short term and long term serious impacts on the health of the patient and sometimes life threatening.

There are relatively high ranking medicinal plants of higher fidelity level in Sheka Zone. They are used to treat humans (204 species), livestock (ten species), and both humans and livestock (52 species) health problems. These high ranking medicinal plants are candidates for further phytochemical profiling in drug research and development.

Medicinal plants with relatively highest use values are considered to be the most used ones. They are considered being under pressure due to over usage which may in the long run can lead to the rarity of the species. Such species need conservation priorities. It was noted that high use diversity index commonly interpreted as the pressure on a given resource arising from use [23]. Therefore, the current study showed that high ranking medicinal plants based on their use diversity values (Table 6) need priority attention for conservation. Hence, there is also a need to establish the direct relationship between the use values of medicinal plants and the actual impact on them arising from harvesting.

## Conclusions

Very high number of medicinal plants recorded from the study area implies that the vegetation of Sheka is good reservoir of medicinally important medicinal plant species. Most of the plant parts used as medicines are the leaves 174 (42.2), root 66 (15.6), and young shoot 32 (7.6%) and the remaining ten plant parts accounting 34% all together. The previous three plant parts play vital role in the life cycle of the plant for continuous functioning. However, over harvesting of these parts have serious effects on the life of the plant. Moreover, the majority of medicinal plant parts 209 (60%) are prepared fresh condition. Hence, traditional healers should frequently rely on fresh plant material. In the meantime, this increases the frequency of use daily or hourly. Therefore, over harvesting can put pressure on locally rare medicinal plant species leading to ultimate extinction. The route of administration and dosage of medicinal plant plants is usually based on haphazard applications except for few well-experienced and knowledgeable traditional healers. Even well-qualified healers are not perfect. The implication of such scenario is that improper use of the medicinal plants can have both short term and long term serious impacts on the health of the patient and sometimes life threatening. There are high ranking medicinal plants that are candidates for further phytochemical profiling in drug research and development.

## Recommendations

Well-known traditional healers of the area should be supported by education, training, and finance to have better knowledge of medicinal plant sustainable use. Chemical profiling of potentially effective medicinal plants (such as *Solanecio mannii*, *Rumex abyssinicus*, and *Prunus africana* all against jaundice) is needed so as to be used as an input for future drug research and development.

## Supplementary information

**Additional file 1.** List of medicinal plant specied collected from the study area: Sheka Zone (Masha, Andracha and Yeki Districts). Key: Cl= Climber, H= Herb, Li= Liana, S= Shrub, T= Tree, Cl= Climber, Coll. No.= Collection Number.

**Additional file 2.** Summary of medicinal plants collected from Sheka Zone and their ethnomedicinal applications KEY: HBT= Habit, T=Tree, S=Srub, H=Herb, PU=Parts used, L=Leaf, R=Root, Bk=Bark, Se=Seed, Fl=Flower, Fr=Fruit, Lx=Latex, Res=Resin, St=Stem, Sht=Shoot, WP=Whole plant, UT=Used to treat, Hu=Human, An=Animals, B=Both, CP=Condition of preparation, Frs=Fresh, Dr=Dry, Lq=Liquid, RA=Route of administration, Ex=External, Dm=Dermal, O=orally, Na=Nasal, Er=Ear, TMC=Total number of medicinal citations. LN=Local names, Sh=Shekinano, Kf=Kefinano, Or= Afan Oromo, Am=Amharic, Sk=Sheko, Mjr=Mejengir, DT=Disease Treated.

**Additional file 3.** Major human and livestock diseases categories in Sheka Zone.

## References

[1] Martin, G.J. *Ethnobotany*. Chapman & Hall, London, UK; 1995.

[2] Balick, M.J. and Cox, P.A.R. Plants, people and culture. The Science of Ethnobotany. Scientific American Library, New York, USA; 1996.

[3] Cotton, C.M. Ethnobotany. Principles and applications. John Willey and Sons, Baffins Lane, Chichester, West Sussex, UK; 1996.

[4] Grenier, L. Working with indigenous knowledge: a guide for researchers. International Development and Research Centre, Ottawa, Canada; 1998.

[5] Cunningham AB (2001). Applied ethnobotany: People, wild plant use and conservation.

[6] WHO. *WHO Monographs on selected medicinal plants, Volume 2*. WHO, Geneva, Switzerland; 2002.

[7] WHO (2002). WHO Traditional Medicine Strategy 2002-2005. WHO, Geneva, Switzerland; 2002.

[8] Jiuzhang, M. and Lei. *A general introduction to traditional Chinese medicine*. CRC Press, Taylor and Francis Group, Boca Raton, London, New York; 2010.

[9] Rai M, Cordell GA, Martinez JL, Marinoff M, Rastrelli L (2012). Medicinal Plants: Biodiversity and drugs.

[10] Osuki PI (2014). African traditional medicine: autonomy and informed consent advanced global bioethics, Volume 3.

[11] Ermias Lulekal, Rondeval Dova, J. Bernaskova, E. Cepkova, J. Zemede Asfaw, Ensermu Kelbessa, Kokosaka, L. and Van Damme, P. Antimicrobial activity of traditional medicinal plants from Ankober District North Shewa Zone, Amhara Region, Ethiopia. Pharm Biol. 2013; Early Online: 1-7. 10.3109/13880209.2013.858362 24392738

[12] Tolosa K, Debela E, Spiridoula AS, Tolera A, Ganga G, Jos GMH (2013). Ethnomedicinal study of plants used for treatment of human and livestock ailments by traditional healers in South Omo, southern Ethiopia. J Ethnobiol Ethnomed.

[13] Wondimu T, Asfaw Z, Kelbessa E (2007). Ethnobotanical study of medicinal plants around “Dheera” Town, Arsi Zone, Ethiopia. J Ethnopharmacol.

[14] Teklehaymanot T, Giday M, Medhin G, Mekonnen Y (2007). Knowledge and use of medicinal plants by people around Debre Libanos Monastery in Ethiopia. J Ethnopharmacol.

[15] Friis, Ib, Sebsebe Demissew and Breugel, P. Atlas of the potential vegetation of Ethiopia. Addi Ababa University press 2011; Sharma Books, Addis Ababa, Ethiopia.

[16] Cochran WG (1977). Sampling Techniques, 3rd edn.

[17] Alexiades MN (1996). Selected guidelines for ethnobotanical research: A field manual.

[18] McClatchey WM, Gollin LX (2005). An ethnobotanical research training workshop in Madagascar. Ethnobot Res Appl.

[19] Hӧft, M., Barik, S.K. and Lykke, A.M. Quantitative ethnobotany. Applications of multivariate and statistical analysis in ethnobotany. Peoples and plants working paper 6. UNESCO. Paris, France; 1999.

[20] Hoffman B, Gallaher T (2007). Important indices in ethnobotany. Ethnobot Res Appl.

[21] Heinrich M (2000). Ethnobotany and its role in drug development. Phyother Res.

[22] Yinger H, Kelbessa E, Bekele T, Lulekal E (2008). Plants used in traditional management of human ailments at Bale Mountains National Park, Southwestern Ethiopia. Journal of Medicinal Plant Research (JMR).

[23] Albuquerque, U.P., Cunha, L.V.F.C., Lucena, R.F.P. and Alues, R.R.N. (eds). Methods and Techniques in ethnobiology and ethnoecology. Springer 2014; science + Business Media, New York, USA.

[24] EFMHACA. Standard Treatment Guidelines for Health Centres 3rd edn, Food, medicine, and health care administration and control authority of Ethiopia (EFMHACA), Addis Ababa, Ethiopia, 2014.

[25] EFMHACA. Standard Treatment Guidelines for Primary Hospitals 2^nd^ edn. Food, medicine and health care administration and control authority of Ethiopia (EFMHACA), Addis Ababa, Ethiopia, 2010.

[26] Elizabeth A, Martin MA (2010). Oxford Concise Medical Dictionary 8th edn.

[27] Giday M, Asfaw Z, Woldu Z (2010). Ethnomedicinal study of plants used by Sheko ethnic group of Ethiopia. J Ethnopharmacol.

[28] Kidane L, Gebremedihin G, Beyene T (2018). Ethnobotanical study of medicinal plants in Ganta Afeshum District, Eastern Zone of Tigray, Northern Ethiopia. J Ethnobiol Ethnomed.

[29] Chekol G (2017). Ethnobotanical study of medicinal plants used against human aliments in Guba Lafto District, Northern Ethiopia. J Ethnobiol Ethnomed.

[30] Yirga G (2010). Assessment of traditional medicinal plants in Endata District, Southeastern Tigray, Northern Ethiopia. African J Plant Sci.

[31] Giday M, Asfaw Z, Woldu Z, Teklehaymanot T (2009). Medicinal plant knowledge of the Bench ethnic group of Ethiopia. An ethnobotanical investigation. J Ethnobiol Ethnomed.

[32] Giday M, Asfaw Z, Woldu Z (2009). Medicinal plants of the Menit ethnic group of Ethiopia: An ethnobotanical study. J Ethnopharmacol.

[33] Hailemariam T, Demissew S, Asfaw Z (2009). An ethnomedicinal study of medicinal plants used by the local people in the lowlands of Kontal Special Wereda, Southern Nations and Nationalities and Peoples Regional State, Ethiopia. J Ethnobiol Ethnomed.

[34] Lulekal E, Kelbessa E, Bekele T, Yinger H (2008). An ethnobotanical study of medicinal plants in Mana Angetu District, Southeastern Ethiopia. J Ethnobiol Ethnomed.

[35] Giday M (2001). An ethnobotanical study of medicinal plants used by the Zay people in Ethiopia. CBM: Skriftserie.

[36] Fiseha Mesfin, Sebsebe Demissew and Tilahun Teklehaimanot (2009). An ethnobotanical study of medicinalplants in Wonago Woreda, SNNPR, Ethiopia. J Ethnobiol Ethnomed 2009; 5 (28). 1746-4269. 10.1186/1746-4269-5-28 PMC2769162 19821994

[37] Hussien Adal. Plant Diversity and Ethnobotany of Borena Sayint National Park, Northern Ethiopia; PhD Theses, Addis Ababa University; 2014.

[38] Tadesse Beyene. Ethnobotany of medicinal plants in Erob and Gulomeheda Districts, Eastern Zone of Tigray National Regional State, Ethiopia. PhD Theses, Addis Ababa University, Addis Ababa, Ethiopia; 2015.

[39] Mirutse Giday. Medicinal plants of the Bench, Meinit and Sheko ethnic groups in Ethiopia with emphasis on use diversity and distribution. PhD Theses, Addis Ababa University, Addis Ababa, Ethiopia; 2007.

[40] Ermias Lulekal (2014). Plant diversity and ethnobotanical study of medicinal plants in Ankober District, North Shewa Zone of Amhara Region, Ethiopia, PhD Theses Addis Ababa University, Addis Ababa, Ethiopia; 2014.

[41] Adefa M, Getaneh S (2013). Medicinal plant biodiversity and local healthcare management system in Chencha District, Gamo Gofa. Ethiopia Journal of Pharmacology and Phytochemistry.

[42] Tilahun Tolossa and Moa Megersa. Ethnobotanical study of medicinal plants used to treat human diseases in Berbere District, Bale Zone of Oromia Regional State Southeast Ethiopia. Hindawi Evidence Based Complementary and Alternative Medicine 2018. 10.1155/2018/8602945 PMC6076952 30105073

[43] Andrade-Cetto A, Heinrich M (2011). From field into the lab: useful approaches to selecting species based on local knowledge. Ethnopharmacology.

[44] Figueroa JI, Fuller L, Abreha A, Hay RJ (1998). Dermatology in southwestern Ethiopia: rationale for community approach. Int J Dermatol.

[45] Kucik CJ, Martin GL, Sortor BV (2004). Common intestinal parasites. Am Fam Physician.

[46] Traore A, Ouedraogo S, Lompo M, Traore S, Some N, Guissou IP (2013). Ethnobotanical survey of medicinal plants used to treat gastrointestinal parasites in human and livestock in four geographic areas of Burkina Faso, (West Africa). Arch Appl Sc Res.

[47] Dawit Abebe, Asfaw Debela and Kelbessa Urga. *Medicinal and other useful plants of Ethiopia*. EHNRI, Addis Ababa, Ethiopia, 2003.

[48] Besufikad E, Bitew D, Messelu Y (2017). Prevalence of intestinal helminthic parasitic infections and associated risk factors among students in Tepi Town Southwest Ethiopia. Science Journal of Public Health.

[49] Tabuti JRS, Lye KA, Dhillion SS (2003). Traditional drugs of Bulamogi, Uganda: Plants, use and administration. J Ethnopharmacol.

[50] Canales M, Hernẚndez T, Caballero J, Romo de vivar A, Avila G, Duran A, Lira R (2005). Informant consensus factor and antibacterial activity of the medicinal plants used by the people of San Rafael Coxcatlan, Puebla, Mexico. J Ethnopharmacol.

[51] Rahaman CH, Karmakar S (2015). Ethnomedicine of Santal Tribe living around Susunia Hill of Bankura District, West Bengal, India: The quantitative approach. Journal of Applied Pharmaceutical Science.

[52] EFMHACA. Standard Treatment Guidelines for General Hospitals 2^nd^ edn. Food, medicine and health care administration and control authority of Ethiopia (EFMHACA), Addis Ababa, Ethiopia, 2010.

[53] Sop TK, Oldeland J, Bognounou F, Schmirdel U, Thiombiano A. Ethnobotanical knowledge and valuation of woody plant species: a comparative analysis of three ethnic groups from the sub-Sahel of Burkina Faso. Environ Dev Sustain. 2012.

[54] Cunningham AB, Shanley P, Laird S (2008). Health, Habitats and Medicinal Plant use.

