# Supplementary material for: An ethnobotanical study of medicinal plants in Sheka Zone of Southern Nations Nationalities and Peoples Regional State, Ethiopia
Source: J Ethnobiol Ethnomed. 2020 Feb 5;16:7. doi: 10.1186/s13002-020-0358-4 (PMC7003439; doi:10.1186/s13002-020-0358-4)
Supplement: Supplementary file 3 — Additional file 3. Major human and livestock diseases categories in Sheka Zone. [file 13002_2020_358_MOESM3_ESM.docx]

# Additional file 3. Major human and livestock diseases categories in Sheka Zone

| SN | Description | Masha | Andracha | Yeki | SZHD |
| --- | --- | --- | --- | --- | --- |
|  | **Human diseases (SZHD*)** |  |  |  |  |
| 1 | All respiratory disease | x | x | x | x |
| 2 | Acute fibril illness (AFI) | x | x | x | x |
| 3 | Pneumonia | x | x | x | x |
| 4 | Mmusculo skeletal system and connective tissue | x | x | x | x |
| 5 | Urinary tract infection | x | x | x | x |
| 6 | Typhoid fever (TFI) | x | x | x | x |
| 7 | Infections of the skin and subcutaneous tissue | x | x | x | x |
| 8 | Other unspecified: skin and subcutaneous tissue | x | x | x | x |
| 9 | Trauma (injury, fracture etc.) | x | x | x | x |
| 10 | Diarrhea (non-bloody) | x | x | x | x |
| 11 | Dyspepsia | x | x | x | x |
| 12 | Helminthiasis | x | x | x | x |
| 13 | Hypertension and related disease | x | x |  | x |
| 14 | Other or unspecified disease of the digestive system | x |  | x | x |
| 15 | Dental and gum diseases | x |  | x | x |
| 16 | Other or unspecified infectious and parasitic diseases | x |  | x | x |
| 17 | Acute bronchitis | x | x | x | x |
| 18 | Epidemic typhus | x |  |  | x |
| 19 | Medical abortion without complication (safe abortion) | x |  | x | x |
| 20 | Diarrhea with blood (dysentery) | x | x |  | x |
| 21 | Other or unspecified disease of the eye and adnexa | x | x | x | x |
| 22 | Asthma | x | x |  | x |
| 23 | Epilepsy | x | x |  | x |
| 24 | Malaria all types |  | x | x | x |
| 25 | Otitis |  | x | x | x |
| 26 | Acute poliomyelitis/Acute flaccid paralysis |  | x |  | x |
| 27 | Diarrhea with dehydration |  | x |  | x |
| 28 | Other unspecified disorders of the genitourinary system |  | x |  | x |
| 29 | Other or unspecified obstetric conditions |  |  | x | x |
| 30 | Factors influencing health status and health services … | x |  | x | x |
| 31 | Human immunodeficiency virus (HIV) |  |  |  | x |
|  | **Livestock diseases (SZHD*)** |  |  |  |  |
| 1 | Bovine pasturulosis/Gorosa/ |  | x | x | x |
| 2 | Black leg/Aba gorba/ | x | x | x | x |
| 3 | Ovine pasturulosis | x | x |  | x |
| 4 | Mastitis/Breast diseases/ | x | x | x | x |
| 5 | New castle diseases (Doro fengil/ |  | x | x | x |
| 6 | Brucellosis/Abortion/ | x | x | x | x |
| 7 | Basbesiasis/tick born diseases/ | x | x | x | x |
| 8 | Rabies | x | x | x | x |
| 9 | Lymph skin diseases | x | x | x | x |
| 10 | African horse sickness/AHS/ | x | x |  | x |

SZHD*= Sheka Zone Health Division (summary of cause reports) (Sheka SZHD, 2013-2015)
